# Integrated multi-omics reveals microbial and metabolic mechanisms driving enhanced fermentation quality in cigar tobacco leaves with exogenous additives

**DOI:** 10.1186/s40643-025-00998-y

**Published:** 2026-01-05

**Authors:** Ping Han, Dongfeng Guo, Mingzhu Zhang, Xuefeng Wu, Dongdong Mu, Yaqi Shi, Rui Zhao, Tianfei Zheng, Xingjiang Li

**Affiliations:** 1 Key Laboratory for Agricultural Products Processing, Anhui Fermented Food Engineering Research Center, School of Food and Biological Engineering, Hefei University of Technology, Danxia Road 485#, Hefei City, 230601 Anhui Province China; 2 China Tobacco Anhui Industrial Co., Ltd., Huangshan Road 606#, Hefei City, 230088 Anhui Province China

**Keywords:** Cigar tobacco leaves, Exogenous additives, Metabolic compounds, Microbial community, Flora structure

## Abstract

Natural exogenous additives (EA) suitable for the tobacco fermentation need to be developed to enhance the fermentation quality and economic value of low-grade cigar tobacco leaves (CTLs). This study analyzed the impacts of three compound Chinese herbal medicine (CHM) on metabolites and microorganisms during CTLs fermentation. The results manifested that EA facilitated the degradation of total sugar, starch and protein, while enhancing the accumulation of reducing sugar in CTLs. Furthermore, EA raised contents of free amino acids (FAAs), while Asp, Glu, Ser and His were found to be key differential FAAs of CTLs. During fermentation, the total contents of volatile flavor components (VFCs) initially increased and then declined. Furthermore, EA contributed to more harmonious compositions of VFCs by promoting the formation of neophytadiene, ketones, esters and aldehydes, as well as facilitating nicotine degradation. According to variable importance in the projection (VIP) > 1 and odor activity value (OAV) > 1, 7 key differential VFCs were identified. EA enhanced positive microbial interactions and led to a more stable and coordinated symbiotic network. Linear discriminant analysis effect size (LEfSe) identified 9 genera as differentially dominant microorganisms in CTLs, which were closely associated with chemical compositions and key differential flavor metabolites. In addition, EA promoted cigar tobacco characteristics (CTCs) by altering bacterial alpha diversity and influencing the assembly of dominant microbial communities. Overall, this study offered theoretical insights into the innovative applications of CHM in CTLs fermentation, and presented new perspectives for enhancing CTLs quality and customizing flavor profiles.

**Graphical abstract:**

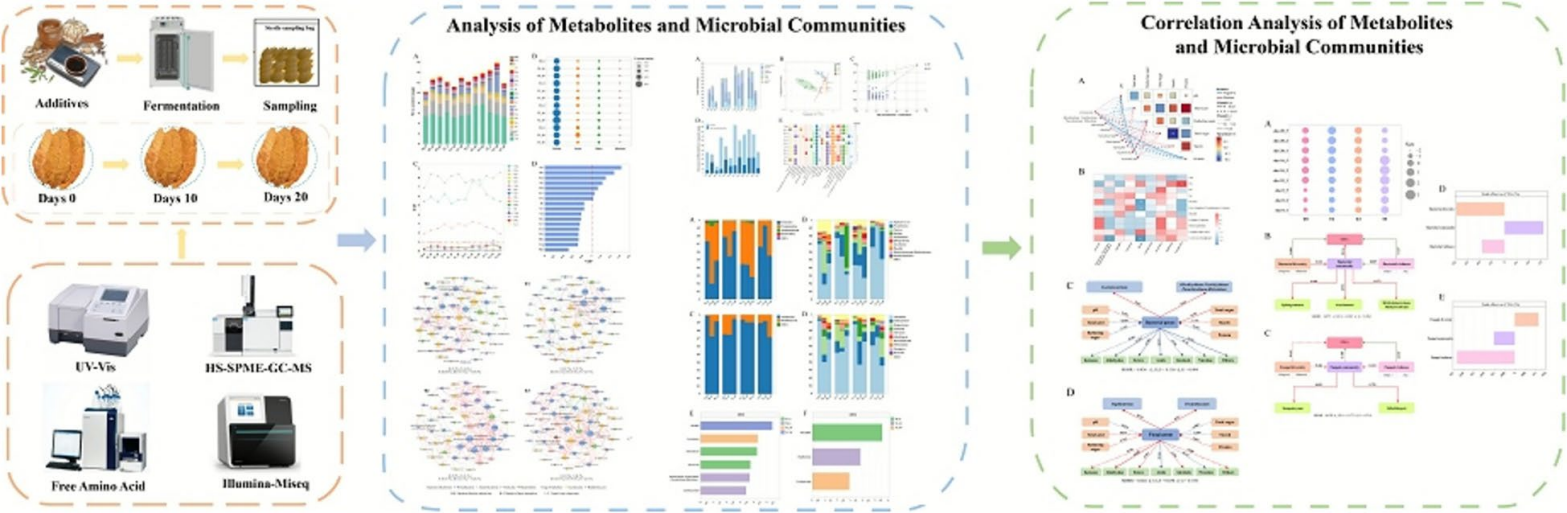

**Supplementary Information:**

The online version contains supplementary material available at 10.1186/s40643-025-00998-y.

## Introduction

Cigars command global attention due to being handmade, possessing distinct aromas, and offering deep flavor profiles (Yang et al. 2022). However, lower-grade cigar tobacco leaves (CTLs) are usually bedeviled by imperfections such as nutrient deficiency and poor blending, which devalue them industrially, result in significant material waste, and hamper the development of cigars. In industrial production, the fermentation process significantly improves the physicochemical and organoleptic properties of CTLs, thereby enhancing their overall quality and compensates for shortcomings in domestic raw materials (Cai et al. 2019; Su et al. 2024). During this process, enzyme-producing microorganisms decompose and transform complex organic substances such as carbohydrates, proteins and starch by secreting enzymes. Meanwhile, aroma-producing microorganisms release nutrients and flavor precursors through metabolism (Guo et al. 2024; Wu et al. 2023; Zhang et al. 2024a). After sufficient fermentation, the contents of volatile flavor compounds (VFCs) rise markedly, distinguishing cigars from vulgaris cigarettes (Fu et al. 2024). Moreover, fermentation also causes significant structural modifications of CTLs to make the leaves softer, whippier and better to roll. Most importantly, fermentation reduces harmful substances, such as nitrates, nitrites, and heavy metals, thereby improving both safety and market competitiveness (Ning et al. 2023).

Currently, artificial fermentation is mostly adopted for the processing of CTLs. Among them, adding exogenous additives (EA) to improve the fermentation quality has gradually become an industry hotspot (Jia et al. 2023). EA including exogenous microorganisms, enzymes and external materials commonly were used by researchers for CTLs fermentation. For instance, co-fermentation with *Bacillus velezensis* A2 and *Bacillus endophyticus* A4 broke down starch, cellulose and proteins, reduced strong odors, elevated aroma levels and boosted sensory quality (Zhang et al. 2024a). Exogenous neutral protease sped up protein degradation, shortened the fermentation time and enhanced the smoothness of CTLs (Zhang et al. 2023b). Besides, natural materials are also incorporated into the CTLs for fermentation. Plant extracts from green tea, pu-erh tea, and tangerine peel, as well as alcoholic additives such as rice wine, have improved the fragrance of CTLs (Li and Zhang 2016). These innovative methods not only optimize traditional fermentation protocols, but also create more opportunities for facilitating the quality and tailoring the taste of CTLs. Considering the innate properties of raw tobacco leaves, it is critical to explore and apply diverse EA from natural plant sources to CTLs fermentation (Hu et al. 2025). These strategies play a significant role in imparting unique flavor characteristics to CTLs and driving technological progress and market growth in the cigar industry. Chinese herbal medicine (CHM) have ancient roots and abundant resources in China. Numerous CHM are contain flavonoids, polysaccharides, amino acids and other bioactive molecules (Jiang 2019; Luo et al. 2019). Licorice root, for example, is widely utilized in the tobacco, food and pharmaceutical sectors (Wei et al. 2013). Its primary components include glycyrrhizin and various flavonoids (Li et al. 2016). Licorice root exhibits anti-inflammatory, antiviral, and antioxidant activities and also enhances immune responses and supports probiotic growth (Zhang et al. 2023c). These properties offer new insights to inhibit mold growth on CTLs during fermentation(Xing et al. 2023). Lingui Ganzao Decoction, detailed in the Treatise on Febrile Diseases, is a classic formula recognized for enhancing metabolic levels, reducing edema and nourishing heart blood. Honeysuckle flower are rich in bioactive compounds including chlorogenic acid, flavonoids, phenolic acids and polysaccharides, which contribute to its well-documented pharmacological properties, particularly antioxidant, anti-inflammatory and antimicrobial activities (Zhou et al. 2025b). The compounds like chlorogenic acid and various flavonoids add the aromatic properties of tobacco products while improving the visual appeal (Hu et al. 2024a, 2025). Honeysuckle flower, chrysanthemum and tangerine peel share a long history in CHMs and often process into wine and tea in folk diets. When combined with licorice root, they provide effects of clearing heat and detoxifying, dispelling cold, warming the middle, strengthening the spleen and soothing the liver. Reishi mushroom is regarded as a nourishing and strengthening fungus that can be used both as medicine and food (Zhao et al. 2016). Meanwhile, dendrobium containing glucose, inulin, fructose and amino acids has a unique aroma and flavor. Therefore, dendrobium is often used as a dietary supplement (Hu et al. 2024b). Research reports that the wine made from dendrobium and reishi mushroom could enhance immunity (Wu et al. 2019). During the fermentation process, polysaccharides participate in Maillard and Caramelization reactions, facilitating the breakdown of macromolecules into smaller carbonyl compounds and key aromatic constituents, thereby enhancing the intrinsic quality of tobacco products (Hu et al. 2025). At the same time, the glucose, fructose and other substances formed by the degradation of polysaccharides are the main energy providers for fermentation microorganisms (Bian et al. 2022), promoting the generation of volatile aroma components through carbon metabolic pathways (Ren et al. 2024).

Collectively, CHM is recognized for its potential to promote the growth of beneficial microorganisms and stimulate the release of bioactive components throughout fermentation (Wang et al. 2019). Adding EA to the fermentation process of CTLs may enhance the overall quality. However, current studies on improving CTLs fermentation quality with CHM are limited. Moreover, the impacts of CHM on chemical components, flavor metabolites and microbial communities in CTLs remain unclear. To address this gap, we selected three CHM mixtures as plant materials: including poria, Guizhi, licorice root and jujube; honeysuckle flower, chrysanthemum, licorice root and tangerine peel; dendrobium and reishi mushroom, which aim to explore the changes in metabolites and microbial community of low-grade CTLs with different CHM during the fermentation. The study analyzes patterns of microbial community succession and the internal connections with metabolites, exploring the role and mechanisms of CHM in microbial succession during CTLs fermentation. The research results offer certain theoretical references for expanding the application of low-grade cigar raw materials and developing new EA.

## Materials and methods

### Preparation of EA

Nine varieties of CHM were procured from the local market in Hefei City. Compound herbal extracts were prepared following methods described in classical texts such as the Treaton Febrile Diseases. The detailed procedures are outlined below:

Extraction of Herb Combination T1: A mixture of 12.5 g of poria, 10 g of Guizhi, 5 g of licorice root, and 3.5 g of jujube was combined with 1600 mL of ultrapure water. The mixture was heated to boiling and then filtered, yielding 300 mL of the first herbal extract.

Extraction of Herb Combination T2: Honeysuckle flower (2.5 g) was mixed with 100 mL of hot water (90 °C) and allowed to stand for 30 min before filtration. The resulting extract was sealed and stored at 4 °C for later use. Chrysanthemum (2 g) was combined with 120 mL of hot water (90 °C), stirred thoroughly, and left to stand for 30 min. After filtration, the extract was sealed with plastic wrap and refrigerated. Licorice root (10 g) was mixed with 100 mL of distilled water and heated at 50 °C for 1 h under continuous stirring. After cooling to room temperature, the solution was sealed and stored at 4 °C. Tangerine peel was ground and sieved through a 40-mesh screen. A total of 100 g of the powder was soaked in 800 mL of water for 30 min, followed by boiling for 30 min and filtration. The residue was boiled again with 600 mL of water for another 30 min and filtered. The combined filtrates were concentrated using a rotary evaporator at 70 °C and 55 rpm to obtain 100 mL of tangerine peel extract (final concentration: 1 g/mL). Extracts of honeysuckle flower, chrysanthemum, licorice root, and tangerine peel were mixed in a 1:1:1:1 ratio and thoroughly combined to obtain 300 mL of the second herbal extract.

Extraction of Herb Combination T3: Dendrobium powder (5 g) was heated with 100 mL of ultrapure water at 80 °C for 2 h. The mixture was filtered after cooling, and the extract was stored under refrigeration. Reishi mushroom (5 g) was mixed with 50 mL of distilled water and extracted at 100 °C for 1 h. The mixture was filtered through a 200-mesh sieve, and the extraction was repeated with the residue under the same conditions. The two extracts were combined, cooled, sealed with plastic wrap, and stored in the refrigerator. Dendrobium and reishi mushroom extracts were mixed in a 1:2 ratio to obtain 300 mL of the third herbal extract.

### The fermentation of CTLs

The raw CTLs used were upper-grade third-level filler leaves (grade: Fi-B-3-Bt-S) without industrial fermentation, that harvested in 2022 in Lincang, Yunnan Province, and classified as Yunxue No. 2. The fermentation was carried out during July 2024. CTLs were isolated according to the standard of approximately 0.10 kg per bundle of tobacco leaves. There were a total of 10 bundles of tobacco leaves in each fermentation group, and a total of 4 fermentation groups. Sterile water was used to make the moisture contents of CTLs to approximately 30%. The prepared EA were sprayed onto the CTLs using a spray gun, ensuring full penetration without droplet formation, while maintaining the moisture content at 30% ± 1%. After humidification, the CTLs were equilibrated in grading frames until the moisture content stabilized between 24 and 25%. The CTLs were put in sterile cloth bags and then stacked in the chamber for fermentation. During the fermentation process, the temperature was set in 35 °C and the humidity was set in 75%. Moisture replenishment was administered timely throughout the fermentation process. Samples were aseptically collected at three fermentation time points (day 0, 10 and 20), representing the unfermented, mid-fermentation, and end-fermentation stages, respectively. A total of 36 samples were collected. The ultrapure water control group and the three herbal treatments were labeled as T0_X, T1_X, T2_X and T3_X, where X denotes the fermentation time (day 0, 10 and 20). The fermentation process and sampling method are illustrated in Fig. S1. CTLs were transferred to sterile bags and reserved for analysing.

### Analyses of chemical components

Before the determination of chemical components, the tobacco leaf samples were pretreated. The CTLs were placed in an oven (DHG-9140A, Shanghai Jinghong Laboratory Equipment Co., Ltd.) at 40 °C and dried until they could be crushable by fingers. The samples were then immediately ground within 2 min, then pass them through a 40-mesh sieve and put them into clean and dry self-sealing bags. The pH value, total acid and protein were measured respectively in accordance with the standards of YC/T 222-2007, GB 12456-2021 and GB/T 5009.5-2016, while total sugar, reducing sugar and starch were detected in accordance with the methods of Wu(Wu et al. 2024b).

### Determination of free amino acids (FAAs)

The amino acid analyzer (S-433D, Germany) was used to detect FAAs (Zhang et al. 2024b; Zhou et al. 2024).

### Determination of VFCs

VFCs were detected by Headspace Solid Phase Micro-extraction Gas Chromatography-mass Spectrometry (HS-SPME-GC–MS; Agilent, USA), referring to the method of the previous authors(Wang et al. 2024b), with adjustments. Sample pretreatment: 0.5 g of undried tobacco sample was weighed into a 20 mL headspace vial, 1 μL (128.75 μg/L) of phenylethyl acetate internal standard solution and 8 mL of saturated sodium chloride solution were added, put into a rotor, equilibrated at 65 ℃ for 20 min (at a rotational speed of 300 r/min), and extracted for 35 min, and then analyzed by using a DVB-CAR-PDMS fiber (50/30 µm, Supelco Inc., Bellefonte, PA, USA) was used to extract volatile flavor components for 35 min, and then the fibers were immediately inserted into a GC–MS equipped with a fused silica capillary column for resolution. Detection chromatographic conditions: inlet temperature 250℃; column HP-5MS (30 m × 0.25 mm × 0.25 μm, J&W Scientific, CA, USA); carrier gas He, no shunt, flow rate maintained at 0.8 mL/min; mass number range 35 ~ 450 m/z; ionization voltage 70 eV; warming procedure: the column temperature of the initial column temperature was 60 ℃, held for 1 min, then increased to 180 ℃ at 3 ℃/min, held for 2 min, and then increased to 260 ℃ at 6 ℃/min, held for 2 min.

Characterization of VFCs was performed by combining the mass spectral information with the NIST 20 standard mass spectral database, and retention indices (*RI*) were calculated:

1 $$ \begin{array}{*{20}c} {{\text{RI}} = 100 \times \left( {\frac{{T_{i} - T_{n} }}{{T_{n + 1} - T_{n} }}} \right)} \\ \end{array} $$

where n is the number of carbon atoms in the n-alkane; *Ti* is the retention time of the compound detected in the sample; and *Tn* and *Tn* + 1 are the retention times of the alkane around the target compound (*Tn* < *Ti* < *Tn* + 1).

The internal standard method was used to quantitatively analyze each substance, and the formula for calculating the content of each substance was as follows:

2 $$ \begin{array}{*{20}c} {A = \frac{{P_{x} \times A_{0} }}{{P_{0} }}} \\ \end{array} $$

where *A* is the flavor substance content (ug/g), *P*_*X*_ is the peak area of the flavor substance, *P*_0_ represents the peak area of the internal standard, and *A*_0_ is the mass concentration (ug/g) formed when the internal standard solution was added to the headspace vial for stabilization.

### DNA extraction and Illumina MiSeq sequencing

Following the method described by (Zhang et al. 2024b), 10 g of tobacco leaves were weighed and ground in liquid nitrogen. Genomic DNA was extracted by using the MagPure Soil DNA LQ Kit (Magan Co., Ltd., Japan). The purity and concentration of the extracted DNA were evaluated using a NanoDrop 2000 spectrophotometer and agarose gel electrophoresis. The DNA was then stored at − 20 °C. Each sample was analyzed in triplicate to ensure biological reproducibility. The V3–V4 hypervariable region of the prokaryotic 16S rRNA gene was amplified using the extracted DNA as a template and primers 338F (5′-ACTCCTACGGGAGGGAGGA-3′) and 806R (5′-GGACTACHVGGGTWTCTAAT-3′). For eukaryotic microorganisms, the ITS1 region of the ITS gene was amplified using primers ITS1F (5′-CTTGGTCATTTAGAGGAAGTAA-3′) and ITS2R (5′-GCTGCGTTCTTCATCGATGC-3′). PCR products were verified via agarose gel electrophoresis. Amplified products were purified with AMPure XP beads, and the resulting DNA served as a template for a second round of PCR. After the second PCR, products were again purified using magnetic beads and quantified using a Qubit fluorometer. DNA concentrations were adjusted for sequencing. Sequencing was performed on the Illumina NovaSeq 6000 platform, generating 250 bp paired-end reads.

Raw sequencing data were obtained in FASTQ format. Trimmomatic (Bolger et al. 2014) was used to preprocess paired-end reads by removing ambiguous bases. After initial filtering with raw sequencing data (Guo et al. 2017), reads were merged using FLASH (version 1.2.11). Operational taxonomic units (OTUs) were clustered at a 97% similarity threshold using UPARSE (version 7.0.1090). Representative OTUs sequences were taxonomically classified using the RDP Classifier with a Bayesian algorithm. Chimeric sequences were identified and removed using the UCHIME algorithm to ensure data accuracy. QIIME (version 1.9.1) was used to evaluate microbial diversity and generate species abundance tables across taxonomic levels. Bacterial sequences were aligned using the SILVA database (release 138), and fungal identification was conducted with the UNITE database (release 8.0).

### Data processing and analysis

Statistical analyses, including one-way analysis of variance (ANOVA) and principal component analysis (PCA), were conducted by SPSS (v.27.0.1), and it was considered that there was a statistical difference when *p* < 0.05. Partial least squares discriminant analysis (PLS-DA) and variable importance in the projection (VIP) were completed by SIMCA (v.14.1). Microbial diversity was assessed by QIIME (v.1.9.1). Linear discriminant analysis effect size (LEfSe) and linear discriminant analysis (LDA) were executed with the cloud platform (https://cloud.majorbio.com/page/tools.html). Microbial co-occurrence networks were constructed using Spearman correlation indices calculated with the “psych” and “corr.test” of R package, and Gephi (v.0.9.3) were employed for network visualization. Partial least square- structural equation (PLS-SEM) model were built and path coefficients like *R*^2^ and *p*-values were estimated by SmartPLS (v.4.1.1.2) (Rönkkö and Evermann 2013). Heatmap were produced by TBtools (v.1.098). Bar charts and curve plots were created by Origin (v.2022). All analytical measurements were performed in triplicate on independent samples, and the results are expressed as the mean ± standard deviation.

### Accession numbers

The raw sequence data reported in this paper have been deposited in the Genome Sequence Archive in National Genomics Data Center, China National Center for Bioinformation/Beijing Institute of Genomics, Chinese Academy of Sciences that are publicly accessible at https://ngdc.cncb.ac.cn/gsa. The BioProject accession numbers PRJCA036435 and PRJCA036436. The corresponding BioSample accessions are subSAM136498 and subSAM136499 respectively.

## Results and discussion

### Changes in chemical composition of CTLs

Chemical components reflect the intrinsic manifestations of the comprehensive quality of CTLs. Chemical indicators such as pH, total acid, reducing sugar, total sugar, starch and protein could basically reflect the quality of CTLs (Zhang et al. 2024c). The results are presented in Table 1. The pH reflected the harmony of the organic and inorganic components, and CTLs with an appropriate pH provide a smooth, harmonious taste and a pleasant smoking experience (Wu et al. 2024a). By day 10 of fermentation, pH in CTLs fermented with EA were markedly higher than those in the T0 group. At day 20 of fermentation, the pH decrease in CTLs fermented with EA ranged from -3.4% to -1.8%, representing a more modest decline compared to that of the T0 group (− 5.9%). Throughout the fermentation process, the total acid content showed a consistent increasing trend in all groups except T3. Upon fermentation completion, the increase in total acid content of CTLs fermented with EA ranged from − 8.1 to 33.2%, which was lower than the 42.4% increase observed in the T0 group. Furthermore, both total sugar and reducing sugar serve as critical biochemical indicators for assessing the quality characteristics of CTLs. Carbohydrates can form diverse aroma substances through multiple chemical reactions (Zhang et al. 2024d). After adding EA, reducing sugar content in CTLs generally increased, while the total sugar content decreased. Compared to T0_20, the reducing sugar content in T1_20, T2_20, and T3_20 increased by 28.6%, 14.3%, and 11.9%, respectively. Compared with T0, EA was more conducive to the accumulation of reducing sugars in CTLs, especially in the T1 group. Starch and protein, as the important macromolecules in CTLs, can negatively impact the sensory quality and burning performance of CTLs. Excessive starch and protein may lead to pungent and bitter taste occur when smoking (Ma et al. 2023). Compared to day 0, the increase in starch content on day 10 was more modest in all EA-treated groups (− 15.6% to 11.8%) than in the T0 group (30.2%). A similar trend was observed for protein content (except for T2). This reduction in the accumulation of starch and protein likely contributed to the milder bitter and astringent taste in EA-treated leaves, possibly due to more vigorous microbial activity degrading these compounds. EA promoted the degradation of starch and protein, thereby contributing to a significant improvement in tobacco leaf quality, highlighting the potential as effective additives for tobacco leaf fermentation (Ma et al. 2023; Ren et al. 2024). Notably, protease enzyme activity in CTLs increases with pH under acidic conditions (Lei et al. 2021). From day 0 to day 10, the pH of CTLs fermented with T1 and T3 increased, indicating that protease activity rose in these groups, resulting in rapid protein degradation. This finding is consistent with the decrease in protein content observed in T1 (− 2.5%) and T3 (− 0.5%) CTLs between day 0 and day 10. As fermentation continued, except for the T1 group, starch and protein contents continued to degrade of tobacco leaves in other groups, reducing pungency and off-flavors and significantly improving the smoking quality. In conclusion, EA induced changes the chemical components of CTLs, resulting in a quality profile distinct from CTLs in T0 group.

**Table 1:** Analysis of chemical constituents of CTLs during the fermentation process

Sample	pH	Total acid/%	Reducing sugar /%	Total sugar/%	Starch/%	Protein/%
T0_0	6.11 ± 0.02^a^	1.44 ± 0.06^ g^	0.43 ± 0.07^d^	13.5 ± 1.0^a^	3.2 ± 0.4^e^	26.7 ± 0.6^ cd^
T0_10	5.79 ± 0.03^de^	1.8 ± 0.1^de^	0.34 ± 0.08^e^	10.8 ± 0.5^b^	4.2 ± 0.2^ab^	28.9 ± 0.9^a^
T0_20	5.75 ± 0.02^e^	2.1 ± 0.1^b^	0.42 ± 0.05^b^	11.4 ± 1.3^b^	3.5 ± 0.1^cde^	28.1 ± 1.1^ab^
T1_0	5.86 ± 0.01^c^	1.9 ± 0.1^bcd^	0.55 ± 0.03^ab^	11.8 ± 0.4^b^	3.8 ± 0.4^abcd^	27.3 ± 0.2^bc^
T1_10	5.88 ± 0.03^c^	1.9 ± 0.1^bc^	0.57 ± 0.02^a^	12.0 ± 0.2^b^	3.2 ± 0.1^e^	26.6 ± 0.6^ cd^
T1_20	5.66 ± 0.01^f^	2.53 ± 0.05^a^	0.54 ± 0.01^abc^	10.9 ± 0.1^b^	4.2 ± 0.2^a^	28.4 ± 0.4^ab^
T2_0	5.99 ± 0.04^b^	1.56 ± 0.03^ fg^	0.46 ± 0.05^ cd^	11.1 ± 0.8^b^	3.8 ± 0.3^bcd^	25.8 ± 0.2^de^
T2_10	5.98 ± 0.04^b^	1.66 ± 0.01^ef^	0.47 ± 0.03^bcd^	9.7 ± 0.7^c^	4.0 ± 0.1^ab^	28.1 ± 0.4^ab^
T2_20	5.88 ± 0.04^c^	2.01 ± 0.02^b^	0.48 ± 0.03^bcd^	11.0 ± 0.6^b^	3.9 ± 0.3^abc^	27.4 ± 0.1^a^
T3_0	5.81 ± 0.03^d^	2.0 ± 0.1^bc^	0.48 ± 0.02^bcd^	11.6 ± 0.6^b^	3.4 ± 0.2^de^	29.1 ± 0.2^bc^
T3_10	6.00 ± 0.01^b^	2.5 ± 0.1^a^	0.41 ± 0.03^de^	9.6 ± 0.5^c^	3.8 ± 0.2^abcd^	29.0 ± 1.7^a^
T3_20	5.57 ± 0.01^ g^	1.8 ± 0.2^cde^	0.47 ± 0.07^bcd^	11.5 ± 0.5^b^	3.5 ± 0.3^cde^	25.3 ± 0.1^e^

The difference of samples was represented by different lowercase letters (one-way ANOVA; *p* < 0.05)

### Changes in FAAs of CTLs

**Fig. 1:**
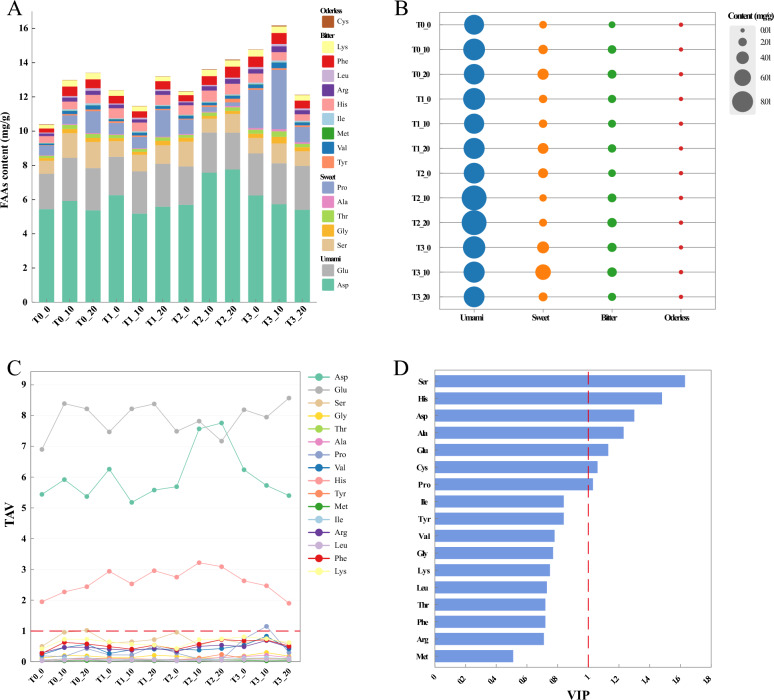
Analysis of FAAs of CTLs during the fermentation process: **A** types and contents of FAAs (mg/g), **B** total contents of four types of flavor FAAs (umami, sweet, bitter and odorless) (mg/g), **C** TAV values of FAAs, **D** VIP values obtained based on PLS-DA model, with the red line indicating TAV ≥ 1

FAAs are important non-volatile metabolites of fermented CTLs and play a dual role in smoke. During the combustion of CTLs, FAAs undergo enzymatic and chemical degradation to yield ammonia (Wu et al. 2024a). On the other hand, FAAs also react with other compounds to generate flavors that are in harmony with aroma and increase the sensory of the smoke (Wang et al. 2024a). 17 FAAs were detected from CTLs (Table S1, Fig. 1A), and divided into 4 groups like umami, sweet, bitter and odorless (Fig. 1B). Umami amino acids, as the predominant FAAs, representing 64.0% of the total FAAs contents. Sweet and bitter amino acids followed, constituting approximately 20.4% and 15.4% respectively, while odorless amino acids made up the smallest proportion at only 0.2%. With the progress of fermentation, the total contents of umami amino acids in T0 and T2 increased and then reached peaks of 8.4 mg/g and 9.9 mg/g at day 10 respectively. In contrast, T1 and T3 showed relatively high levels of umami amino acids at day 0, the unfermented stage. For sweet amino acids, the T3 group performed the best, ranging from 2.3 to 5.5 mg/g with a peak on day 10. Additionally, the concentrations of bitter amino acids increased in T0 and T2, while decreasing in T1 and T3. However, The content of bitter amino acids remained relatively low throughout the fermentation. Judging from the above results, EA amended the FAAs contents of unfermented CTLs (day 0), which may be attributed to the active substances carried by EA (Jiang 2019; Luo et al. 2019). During the subsequent fermentation process, proteins were degraded by proteases into various amino acids. These amino acids and their metabolites extensively participated in multiple amino acid metabolic pathways. For example, secondary metabolites such as benzaldehyde, phenylacetaldehyde, and benzyl alcohol, which were generated via the phenylalanine biosynthesis pathway, contributed significantly as key aroma compounds in CTLs (Chen et al. 2025).

To identify FAAs with significant contributions to CTLs, the taste activity values (TAV) in different fermentation groups were calculated. TAV is calculated as the ratio of the concentration of the amino acid to its threshold. The FAA is considered a taste-active amino acid that significantly contributes to the overall taste when its TAV is greater than 1 (Wang et al. 2024a). The TAV of Asp, Glu and His were all > 1 across all samples (Fig. 1C), indicating their significant sensory contributions. These amino acids imparted umami and bitter characteristics to the CTLs, and the TAV of Asp and Glu occupied absolute dominant positions. Asp and Glu defined the particularly prominent umami style of Yunxue No. 2 CTLs under industrial fermentation. The CTLs with T2 showed a higher contents of umami amino acids, with a progressive increase with the progress of fermentation. Therefore, it was speculated that T2 shaped stronger umami characteristics of CTLs. Furthermore, PLS-DA was performed on the FAAs data matrix. The model demonstrated high robustness with *R*^2^*X* = 0.73, *R*^*2*^*Y* = 0.98, and *Q*^2^ = 0.94. The permutation test also proved the the goodness of model validity of fit with *R*^2^ = 0.27 and *Q*^2^ =  − 1.05. With the selection criteria of VIP ≥ 1.0 and *p* < 0.05, 7 amino acids were considered as the most discriminating variables (Fig. 1D). Based on the results of TAV and VIP, Asp, Glu, Ser and His were selected as the key amino acids to set apart from CTLs in different groups. The research of (Zhang et al. 2024c) also verified that umami amino acids (Glu and Asp) are the most distinguishable FAAs of CTLs.

### Changes in VFCs of CTLs

**Fig. 2:**
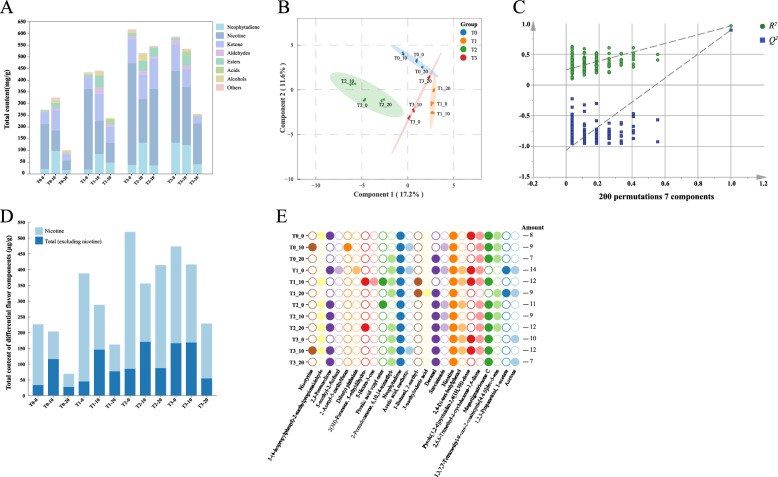
Analysis of VFCs of CTLs during the fermentation process: **A** Bar chart of the contents of VFCs. PLS-DA were applied to analyse VFCs of CTLs: **B** the distribution point map, **C** permutation test, **D** bar chart of the contents of differential VFCs (VIP > 1, *p* < 0.05), **E** distribution map of differential flavor substances in the sample, solid circles indicated that they were detected in the sample, and hollow circles indicated that they were not detected

HS-SPME-GC-MS was used to analyse the VFCs of CTLs and 53 VFCs were detected (Table S2). The kinds of VFCs were semblable in CTLs under different groups, though their concentrations varied. Except for nicotine and neophytadiene, the VFCs were classified into six categories based on the molecular structures (Fig. 2A), including 23 ketones, 7 aldehydes, 9 esters, 4 alcohols, 4 acids and 4 others. EA, containing active substances like sugars and acids, directly increased the contents of VFCs. On day 0 of fermentation, EA significantly increased the total VFCs in CTLs. However, in all groups, unfermented CTLs had excessively high nicotine levels, low neophytadiene content, and an absonant distribution of VFCs. This suggested that unfermented CTLs had a stronger bitter taste, an unbalanced smoke flavor, and overall poorer quality. After 10 days of fermentation, although the total amount of VFCs decreased, the process generally promoted the formation of neophytadiene, ketones, esters, and aldehydes, especially leading to the rapid degradation of nicotine. The significant reduction in nicotine contributed to the overall decrease in flavor compounds in CTLs, which weaken the irritability of CTLs (Zhang et al. 2018). Neophytadiene, as the component with the highest content of VFCs in CTLs, directly affected the taste, aroma and the formation of other aromatic compounds (Yan et al. 2022; Yun et al. 2023). Besides, ketones, as the most numerous and abundant compounds excluding neophytadiene and nicotine, were the main flavor components in CTLs (Wu et al. 2024b). Ketones with abundant contents, such as *β*-ionone and solanone, provided CTLs with obvious delicate fruity and floral characteristics. Esters contributed to sweet and fruity flavors (Wang et al. 2024b), while aldehydes imparted woody and slightly oily aromas (Xu et al. 2022). These flavor substances were more abundant in the EA-fermented CTLs, and were proven to be of great significance for enhancing the aroma of CTLs in previous research (Hu et al. 2024a). Furthermore, the sugars, acids, alcohols and active compounds of EA not only directly increased the content of VFCs but also sped up microbial metabolism to promote the production of aromatic compounds. These consequences stressed the necessity of industrial fermentation for enhancing the fermented quality of CTLs. When the fermentation was completed, CTLs with T2 still maintained a relatively high contents of VFCs, but others showed varying degrees of flavor compounds loss. The results indicated that excessive fermentation hindered the accumulation of VFCs (Hu et al. 2023).

Results of PLS-DA revealed that CTLs in different groups could be clearly distinguished (Fig. 2B). Within the same fermentation group, the flavor compositions of the samples under different fermentation times also varied, indicating that both fermentation time and EA influenced the flavor compositions simultaneously. Except for T2_20, the samples at day 20 were relatively clustered, suggesting that there were certain similarities in the flavor compositions of CTLs at the final fermentation stage. To evaluate model validity, 200 permutation tests were conducted and the results confirmed its good fit (Fig. 2C). The differences in the composition of VFCs during the fermentation process in different groups were further verified. With *p* < 0.05 and VIP ≥ 1.0 as the standard, a total of 24 significantly different VFCs were screened out (Table S2). These included nicotine, neophytadiene, 8 ketones, 4 esters, 3 aldehydes, 2 acids, 1 alcohol and 4 others. Ketones were the dominant flavor compounds in CTLs and the main contributors to flavor differentiation. Compared with the EA groups, CTLs in T0 group showed fewer contents and a lower types of differential VFCs **(**Fig. 2D and E), which indicated a feeble flavor characteristic. EA promoted the formation and altered the abundance of differential VFCs. The total amounts of differential VFCs of CTLs fermented with EA gradually declined over fermentation, that were correlated with the significant decrease in nicotine. Excluding nicotine, differential VFCs of CTLs first increased and then declined during fermentation peaking on day 10 respectively (Fig. 2D). Furthermore, as shown in Fig. 2E, CTLs fermented with EA amendments showed a greater diversity of differential volatile flavor compounds compared to the T0 group. The elevated levels of these compounds of CTLs in the EA groups contributed to particular flavor profile.

VFCs are generally regarded as major contributors to flavor characteristics while odor activity values (OAV) ≥ 1 (Wang et al. 2024b). The OAVs of 53 VFCs were calculated, and 22 compounds showing the OAV ≥ 1 as detailed in Table S3. According to Table S3, nicotine, *β*-ionone, 4,7,9-megastigmatrien-3-one A, B, D, benzeneacetaldehyde, nonanal, decanal and cedrol exhibited exceptionally high OAV exceeding 500. This demonstrated how crucial the role they played in determining the flavor of fermented CTLs. Nicotine, solanone, geranylacetone and 4,7,9-megastigmatrien-3-one, as key VFCs of tobacco, all showed OAV > 1. Furthermore, Additionally, when PLS-DA and OAV were combined, seven important VFCs were in charge of the aromatic variations among the CTLs (*p* < 0.05, VIP ≥ 1 and OAV ≥ 1), including nicotine, 2,6,6-trimethyl-2-cyclohexene-1,4-dione, decanal, 5-methyl-2-furfural, dibutyl phthalate, 3-methylvaleric acid and 2,4-di-tert-butylphenol. The 2,6,6-trimethyl-2-cyclohexene-1,4-dione contributes fruity and citrus notes (He et al. 2025). Decanal imparts sweet and fatty aromas (Zhuang et al. 2020). 5-methyl-2-furaldehyde, a key product of the Maillard reaction, is responsible for the distinctive caramel-like sweetness in CTLs (Jiang 2025). 3-methylvaleric acid is associated with an acidic, cheese-like odor (Cai et al. 2024; Zhuang et al. 2020). Overall, the varying abundances of these VFCs across different EA-fermented CTLs led to distinct flavor profiles.

### Analysis of microbial community diversity of CTLs

**Fig. 3:**
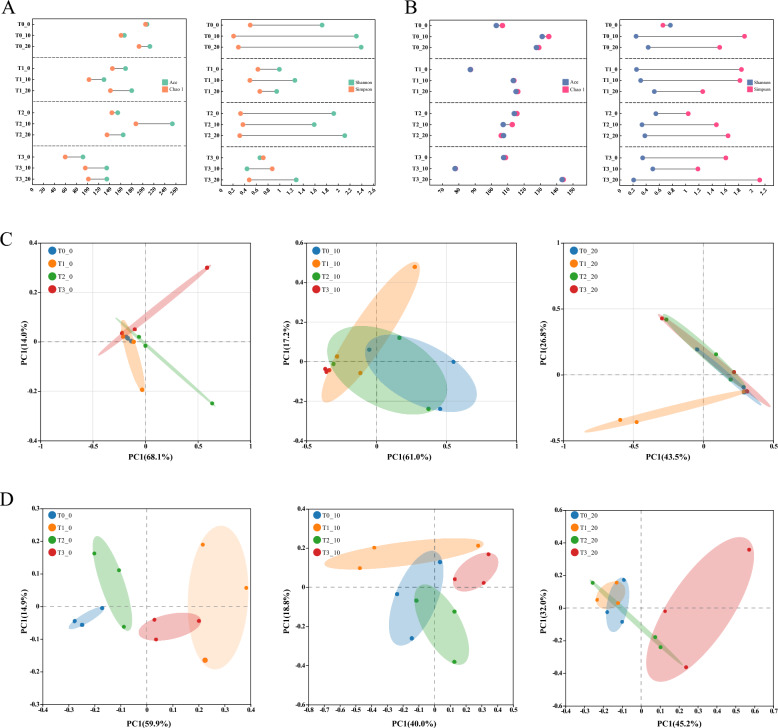
Analysis of alpha diversity of microbial community in CTLs: **A** bacterial community, **B** fungal community. PCoA analysis of fermented CTLs based on different EA groups: **C** bacterial community, **D** fungal community

The microbial communities in CTLs during fermentation under different EA treatments were analyzed using high-throughput sequencing. All samples exhibited good coverage (Fig. S2), confirming that the sequencing depth was sufficient to saturate microbial diversity and capture the majority of microorganisms. Alpha diversity of CTLs from different EA treatments was investigated using the Chao1, Ace, Shannon, and Simpson indices. The results for bacterial and fungal community diversity are shown in Fig. 3A and B, respectively. It was observed that the trends for groups T0 and T1 were relatively similar, while T2 and T3 also showed consistent trends with each other. Specifically, the richness of bacterial communities in CTLs from T0 and T1 groups reached its lowest point at day 10, but their diversity was higher. In contrast, the bacterial richness in T2 and T3 CTLs peaked at day 10, while their diversity was lower. Notably, the bacterial richness of CTLs in T2_10 was particularly prominent. For fungal communities, CTLs in the T0 and T1 groups exhibited higher diversity at day 10, whereas the T2 and T3 groups showed higher diversity at day 20. Overall, the microbial community diversity in CTLs fluctuated considerably during fermentation, suggesting relatively intense community succession. Furthermore, Principal Coordinates Analysis (PCoA) based on Bray–Curtis distance was used to reveal the distribution of microbial communities among different fermentation groups at the same time point (Fig. 3C and D). For bacterial communities, from day 0 to day 20, the CTLs from different groups remained relatively clustered. By day 20, the T0, T2, and T3 groups almost completely overlapped, indicating that EA had no pronounced effect on bacterial diversity throughout the fermentation process. For fungal communities, however, the CTLs from different fermentation groups transitioned from a dispersed to a more clustered pattern. This suggests that the addition of EA rapidly altered the fungal diversity in CTLs, but as fermentation progressed, these differences gradually diminished. Nevertheless, it is undeniable that certain differences persisted between the EA-treated groups and the naturally fermented CTLs (T0).

### Analysis of microbial community composition of CTLs

**Fig. 4:**
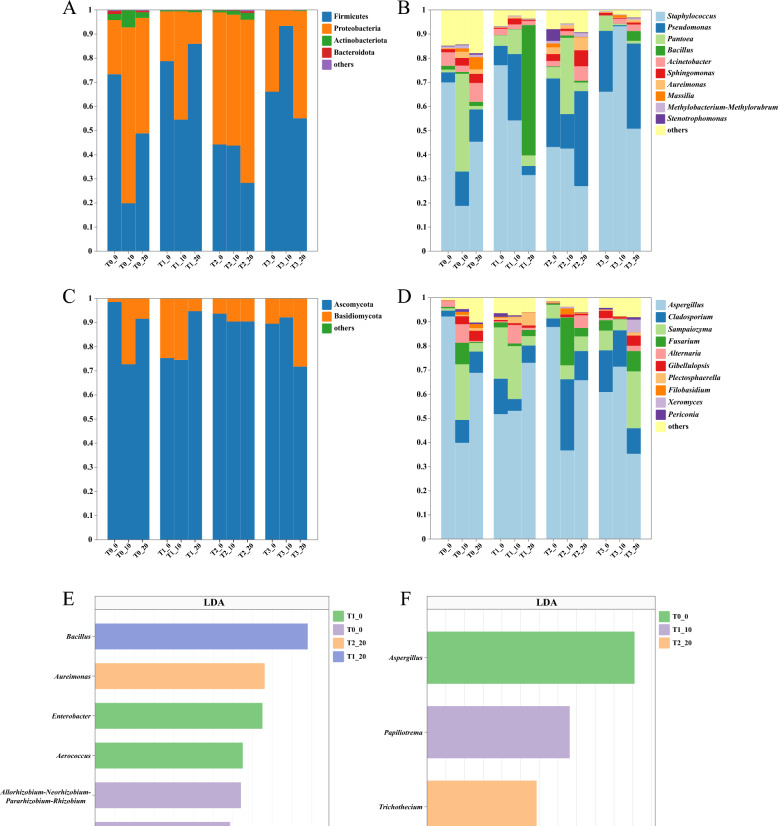
Analysis of the microbial community compositions of CTLs: stacked bar stack diagrams of bacteria (**A**) and fungi (**B**) at the phylum level, stacked bar stack diagrams of bacteria (**C**) and fungi (**D**) at the genus level. LEfSe analysis at the genus level of the characterized microorganism, bacteria (**E**) and fungi (**F**)

Bacteria and fungi act synergistically in degrading macromolecules such as carbohydrates to influence the generation and accumulation of flavor metabolites during CTLs fermentation. Taxonomic analysis was performed to determine the community compositions of CTLs. 4 bacterial phyla and 2 fungal phyla were identified based on a relative abundance > 1%. More than 99% of microorganisms in bacterial communities were assigned to Firmicutes, Proteobacteria, Actinobacteriota and Bacteroidota (Fig. 4A), and this phenomenon was in line with the bacterial makeup of domestic CTLs (Wang et al. 2024b). The two most dominant of these, Firmicutes and Proteobacteria, experienced dynamic changes throughout fermentation. During the fermentation of CTLs, Firmicutes and Proteobacteria both play essential roles in carbon degradation by converting starch, cellulose, and pectin into maltose, fructose, and glucose (Costa et al. 2020). Compared to T0 group, relative abundance of Firmicutes significantly increased in CTLs fermented with EA, while Proteobacteria showed a declining trend. A total of 33 bacterial genera in terms of relative abundance were identified, and the top 10 were shown in Fig. 4B. After fermentation, *Staphylococcus* occupied a larger proportion in EA groups and was the most dominant genus (16.7–93.2%). *Staphylococcus* is known to generate lipases and proteases, that assist in the formation of VFCs. (Pei et al. 2025). Previous research have demonstrated that *Staphylococcus* in CTLs fermented with spice extracts exhibited a consistent succession pattern, potentially promoting the progression and efficiency of the CTL fermentation process (Hu et al. 2025). *Pseudomonas* exhibited strong proteolytic activity and was also the dominant genus (0.5–35.7%) (Si et al. 2023). The total abundance of *Staphylococcus* and *Pseudomonas* was higher in CTLs fermented with EA than that in the T0 group. This phenomenon further supported the previous inference that EA enhanced microbial activity and promoted the degradation of starch and protein, resulting in lower protein and starch contents (Table 1). Other dominant genera also play important roles in flavor compounds in CTLs. *Bacillus*, for instance, encourages the breakdown of carotenoids For example, *Bacillus* promotes the degradation of carotenoids into small aromatic compounds through glycosidase production (Huang et al. 2022). *Sphingomonas* can metabolize chlorogenic acid, and the intermediate product is an aromatic organic acid that serves as important aromatic precursors (Wang et al. 2024b). The relative abundance of *Pantoea*, which can decompose tannins and reduce bitterness, was especially prevalent of CTLs in T0_10 and T2_10 (Zhang et al. 2023a). Both *Sphingomonas* and *Pseudomonas* degrade nicotine. In particular, *Pseudomonas* can utilize nicotine as a special source of carbon and nitrogen (Li et al. 2020; Wang et al. 2023). Similar successional patterns in CTLs were shown by these two genera, implying that they might have similar niches and survival methods during the fermentation of CTLs.

In the fungal communities, Ascomycota and Basidiomycota were the dominant phyla because of accounting for over 95% of the total relative abundance (Fig. 4C). Among them, the relative abundance of Ascomycota consistently represented more than 70%. Thirty fungal genera with relative abundance > 1% were identified and the top 10 genera shown in Fig. 4D. *Aspergillus* was the predominant genus in all samples, with relative abundance ranging from 35.3 to 92.2%. However, the dominance of *Aspergillus* decreased in the later stages of fermentation. In the meantime, the relative abundance of *Cladosporium* and *Sampaiozyma* increased. *Aspergillus* plays a dual role in enhancing CTLs flavor positively by degrading organic matter, particularly sugars and proteins (Zhang et al. 2024e). On the other hand, excessive proliferation of *Aspergillus* during fermentation may lead to surface hyphae formation, resulting in mold growth that damages CTLs quality (Chen et al. 2019). To solve this problem, workers often adjust the environmental temperature and humidity to reduce the abundance of mold in fermentation. Notably, CTLs with T2 maintained lower *Aspergillus* during d10 to d20 compared with T0, which suggested that T2 may inhibit mold development on CTLs due to its unique formulation. According to previous research, honeysuckle flowers have antifungal properties that prevent the growth of mold (Bahk and Marth 1983). Chrysanthemum shows demonstrate strong antifungal activity against *Aspergillus niger* and *Aspergillus flavus* (VA et al. 2019) and tangerine peel inhibits *Aspergillus flavus* (Chen et al. 2022; Wu et al. 2014). These ingredients may work in concert to suppress mold activity in CTLs. *Cladosporium* is frequently found in CTLs and is well-known for generating important enzymes such as amylases and proteases that are essential for boosting sweet and roasted aromas (Zhang et al. 2021, 2025). *Sampaiozyma* is a dominant genus in Dominican and Indonesian CTLs and closely associated with sweet and sour aromatic characteristics (Wang et al. 2024a). During fermentation, it contributes to forming aroma compounds like esters and alcohols by metabolizing sugars and other organic substances. Therefore, the polysaccharides, amino acids, flavonoids, and other components of EA served as metabolic substrates during microbial growth and metabolism. This facilitated the degradation and transformation of macromolecules within CTLs, ultimately enhancing the aromatic properties. Interestingly, despite differences in relative abundance, the dominant fungal genera were consistent across CTLs in different groups. This indicated that the changes in the fermentation microenvironment caused by EA drove the same changing trend of the dominant microbial genus and colonized CTLs, improving the fermentation quality of CTLs.

The synergistic fermentation with EA undoubtedly altered the microbial compositions in CTLs. The Venn diagram clearly visualized the similarities and unique species compositions of CTLs (Fig. S3). A total of 40 bacterial and 44 fungal genera were shared in different groups. As fermentation progressed, the figures of endemic bacterial genera in T0 and T1 groups decreased and then increased, while the number of endemic fungal genera showed a trend of first increased and then decreased. In contrast, the unique genera in the T2 and T3 groups exhibited trends opposite to those in T0 and T1. This trend also implied that T0 and T1, as well as T2 and T3 had comparable microbial succession patterns throughout fermentation. To gain a greater awareness of the microbial community differences in CTLs, LEfSe analysis were performed to distinguish statistically significant markers in different groups. The results are shown in Table S4 (LDA > 2.5, *p* < 0.05). 22 bacterial and 11 fungal biomarkers were distinguished, while bacterial community differences were more pronounced than fungal differences. Combining with relative abundance > 1%, a total of 6 bacterial and 3 fungal genera were distinguished as differential dominant genera in tobacco leaves (Fig. 4E and F). *Bacillus* breaks down alkaloids and raises the concentration of flavor compounds to improve the quality of cigar (Zhou et al. 2025a). *Aureimonas* is an endophyte that essential to preserving the ecological stability of CTLs (Zhang et al. 2019). *Papiliotrema* unites with *Bacillus subtilis* shows potent antimicrobial properties and antagonistic activity against fusarium wilt and wheat crown rot (Liu et al. 2021). In conclusion, the quality differentiation of CTLs in different groups was probably driven by these 9 differential dominant genera.

### Analysis of the co-occurrence network structure of CTLs

**Fig. 5:**
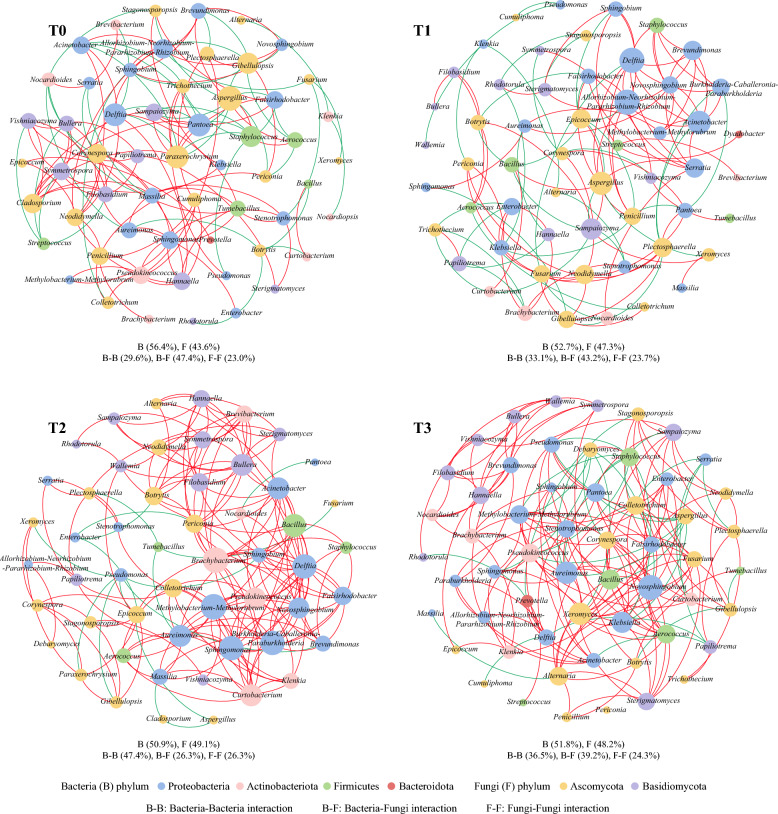
Based on the symbiotic networks of dominant genera (relative abundance > 1%) during the fermentation process of CTLs. Nodes with same color indicated belonging to the identical classification level, which sizes were represented by its connection degree and reveals the importance of the node in the symbiotic network. The red line and the blue line respectively indicated that there are positive and negative interactions between the nodes

Microorganisms typically exist in complex communities and form tight relationships with each other. The symbiotic network illustrated the interactions between microbial communities of CTLs in different fermentation groups. Genera with relative abundance > 1% were selected, and microbial symbiotic networks of CTLs in different groups were constructed based on prominent correlations (r >|0.5|, *p* < 0.05) (Fig. 5). The proportion of bacterial nodes in the symbiotic network of CTLs decreased in the EA groups (50.9–52.7%) compared to the T0 (56.4%), while the proportion of fungal nodes increased. This discovery revealed that EA enhanced the role of fungi in maintaining the symbiotic network while reducing the role of bacteria. The nodes in the network were classified into four bacterial and two fungal phyla. Among them, Proteobacteria was the regnant bacterial phylum (29.8–34.6%) of the total nodes, while Ascomycota was the regnant fungal phylum (29.1–31.6%). The microorganisms of Ascomycota and Proteobacteria occupied more central positions with more connections between nodes to other microorganisms in CTLs, playing a primary role in maintaining network stability. The positive correlations between nodes showed that 64.4%, 65.3%, 91.4% and 81.8% of total interactions in the T0, T1, T2 and T3 groups respectively. This showed that dominant microorganisms in CTLs tend to form cooperative relationships rather than competitive ones, while CTLs fermented with EA particularly T2 and T3 promoted the development of these relationships. Notably, interactions between bacteria and fungi were weakened and interactions between bacteria and bacteria as well as between fungi and fungi were strengthened in CTLs fermented with EA compared to T0 group. Additionally, *Staphylococcus* mostly exhibited competitive relationships with other genera in T0 and T3 groups, which was in accordance with the researches of Li (Li et al. 2023). *Staphylococcus* was better adapted to the surface environment of CTLs and enabled to rapid growth by utilizing available nutrients, while its growth and metabolism may inhibit those of other dominant genera.

Topological structure refers to the geometric configuration and connection patterns of symbiotic networks, which represent the dynamic changes in the microbiome’s symbiotic structure and adjustments in its ecological functions. The quantity of interactions both within and between taxa is reflected in the number of nodes and edges. The average degree measures network complexity and the graph density indicates network integrity. The average clustering coefficient reflects node clustering and the average path length represents the efficiency of material, energy and information transfer between species. The topology of the microbial network is presented in Table 2. Compared to T0, the number of nodes decreased in EA groups, but the amounts of edges significantly increased in T2 and T3 groups, along with higher average degrees, graph densities, clustering coefficients and shorter average path lengths. In contrast, the T1 group exhibited an opposite trend. These findings demonstrated that the microbial ecological network in tobacco leaves of the T2 and T3 groups became more complex and formed tight group structures with more nodes. The scale and stability of the community structure significantly increased (Ji et al. 2025). Meanwhile, the efficiency of microorganisms in the community in transferring substances, energy and information is relatively high which made it less susceptible to environmental influences. This may contribute to greater steadiness of the community structure but the network in the T3 group was incompact. These modifications were a reflection of the adaptive modifications in the microbiome network of CTLs fermented with EA, which led to different patterns of microbiota interaction. The introduction of T2 and T3 not only changed the microbial community compositions but also enhanced the stability of the community structures.

**Table 2:** Topological characteristics of microbial networks in CTLs

Topological characteristic	Node	Edge	Average degree	Graph density	Average clustering coefficient	Average path length
T0	57	135	4.74	0.085	0.45	3.30
T1	55	118	4.29	0.079	0.37	3.42
T2	53	186	7.02	0.14	0.47	2.83
T3	56	181	6.50	0.12	0.51	2.93

### Correlation analysis of microbial communities and quality characteristics of CTLs

**Fig. 6:**
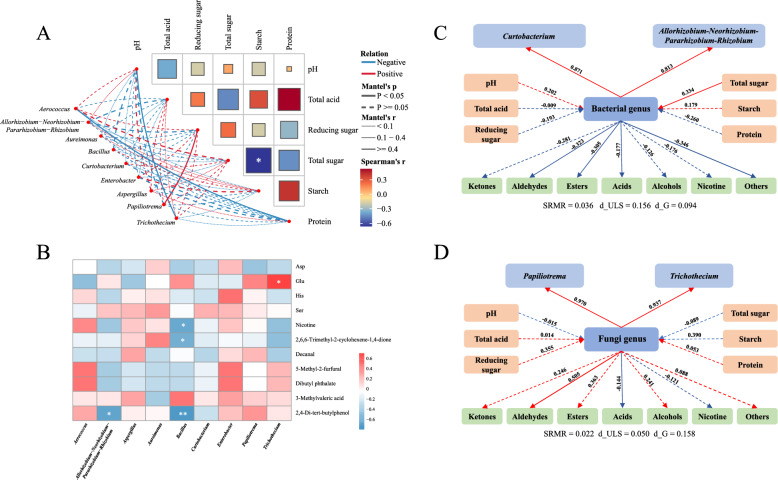
**A** Mantal-test analysis of differentially dominant microorganisms and chemical components. The heat map represented the intra-group correlation of chemical components, and the network diagram represented associations between differential dominant microorganisms and chemical components. **B** Spearman correlation analysis based on differential dominant microorganisms and key differential flavor metabolites. * and ** indicate significant differences at *p* < 0.05 and *p* < 0.01 respectively. PLS-SEM analysis of differentially dominant genera, chemical composition and VFCs: **C** bacteria, **D** fungi

The chemical components, flavor metabolites and microbial communities in CTLs interact complexly, influenced each other and drove fermentation of CTLs. Mantal test was used to analyze correlations between differential dominant microbiota and chemical components to elucidate the impact of dominant differential microorganisms on CTLs (Fig. 6A). Among the chemical components, starch and total sugar exhibited a significant negative correlation (*p* < 0.05). Reducing sugar displayed the positive correlation with *Papiliotrema* (r = 0.58, *p* < 0.05). pH was adversely relevant with *Trichothecium* (r =  − 0.62, *p* < 0.05) and protein adversely associative with *Aerococcus* (r =  − 0.75, *p* < 0.01). In summary, changes in chemical parameters are inseparably connected to microbial community shifts during CTLs fermentation. As shown in Fig. 6B, the differential dominant microbiota were correlated with four key amino acids and seven key aroma substances. Above the four key amino acids, *Trichothecium* exhibited a prominent positive correlation with Glu (*p* < 0.05). Ser exhibited positive interactions with most dominant genera. Interestingly, *Aspergillus* showed a negative correlation with two umami amino acids (Asp, Glu), indicating that its reproduction inhibited the aroma of umami amino acids in fermented CTLs. The deduction aligned with the lower relative abundance of *Aspergillus* and the more prominent umami amino acids in the T2 group (Figs. 1B, 4D). Overall, during fermentation, changes in the abundance of differential dominant microorganisms drive more intense amino acid metabolism (Chen et al. 2025), which affects FAAs contents and aroma expression, ultimately determining the quality of CTLs. In contrast, key differential VFCs exhibited the significant adverse connections with differential dominant microorganisms. For example, *Bacillus* was negatively correlated with nicotine, 2,6,6-trimethyl-2-cyclohexene-1,4-dione (*p* < 0.05), while *Allorhizobium-Neorhizobium-Pararhizobium-Rhizobium* was negatively correlated with 2,4-di-tert-butylphenol (*p* < 0.05). The correlation analysis results showed that the microbial community was tightly relevant to key differential flavor metabolites, and interactions between microorganisms pushed fermentation and influenced flavor of CTLs.

PLS-SEM models were applied to further explore the effect of key differential microorganisms and chemical components on flavor metabolites in CTLs. The model was considered acceptable and valid when SRMR < 0.08, d_ULS < 0.95, and d_G < 0.95(Rönkkö and Evermann 2013). As no valid model could be established with FAAs, the study assessed the correlations among chemical components, dominant differential microbial genera and VFCs (Fig. 6C and D). The results manifested that the model provided rational predictive consequences. Total sugar had the strongest effect on bacterial genera (*p* < 0.05) but a negative effect on fungal genera. Among the eight key differential bacterial genera, *Allorhizobium-Neorhizobium-Pararhizobium-Rhizobium* and *Curtobacterium* had *R*^2^ of 0.871 and 0.813, explaining a large portion of bacterial community variation. For fungal communities, *Papiliotrema* and *Trichothecium* accounted for most of the changes, with *R*^2^ of 0.97 and 0.94 respectively. Moreover, dominant differential microorganisms directly impacted VFCs. Bacterial were mostly negatively associative with VFCs, whereas fungal were primarily positively correlated. Bacterial genera showed significant negative correlations with aldehydes, esters, acids and others (including neophytadiene) (*p* < 0.05), whereas fungal genera had a significant positive effect on aldehydes and a negative effect on acids (*p* < 0.05). Total sugar, as the most influential chemical component, affected both bacterial and fungal genera positively and directly influenced flavor metabolites. In particular, the total sugar content was generally lower in CTLs fermented with EA than in the T0 group, despite fluctuations during fermentation. This modification further encouraged the formation of flavor metabolites especially aldehydes, while mildly favorably affecting fungal genera. Furthermore, these changes significantly suppressed variation in some bacterial genera. Therefore, by adjusting the total sugar content during fermentation, EA may change the microbial community and flavor quality of CTLs.

**Fig. 7:**
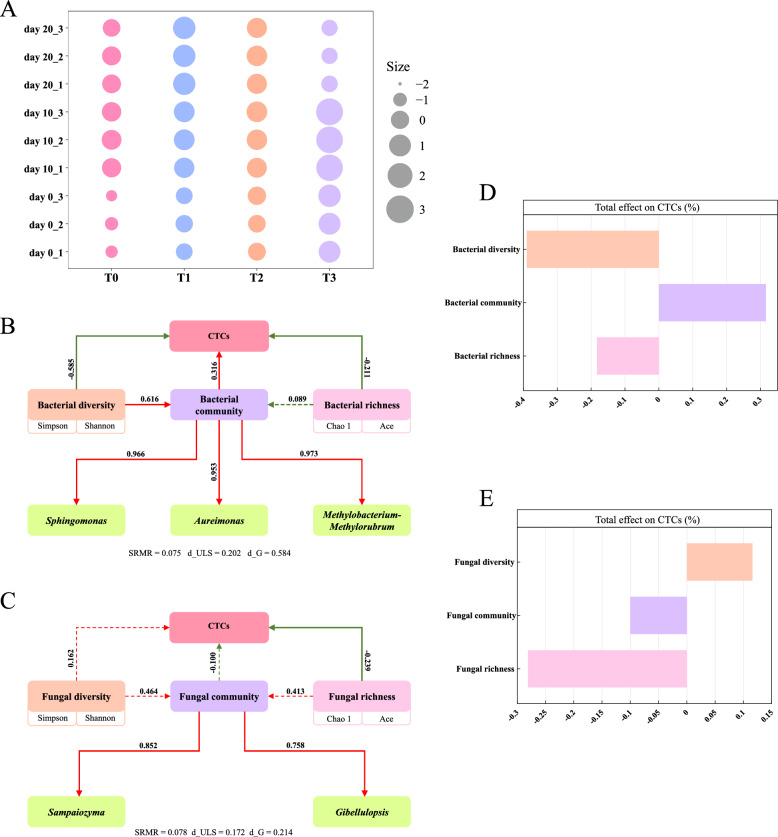
**A** The comprehensive scores of samples were calculated by using the principal component analysis method. The PLS-SEM model revealed the impacts of the alpha diversity and dominant communities of bacterial (**B**) and fungal (**C**) communities on CTCs, and the coefficients on the path represent the variance of the explanations. Red arrows indicated positive correlations and green arrows indicated negative correlations. Solid lines indicated prominent correlations (*p* < 0.05), while dashed lines indicated insignificant correlations (*p* ≥ 0.05). The influence of the standardized total effect of alpha diversity and dominant community compositions of bacteria (**D**) and fungi (**E**) on CTCs

PCA simplifies the evaluation by reducing numerous indicators into a few independent components (Aboytes-Ojeda et al. 2016). PCA was applied to six chemical components, four categories of FAAs, and eight categories of VFCs. Weakly correlated variables, such as pH, reducing sugars, protein, odorless amino acids and other VFCs, were removed through iterative analysis. The final model (KMO = 0.51, *p* < 0.001) indicated sufficient inter-variable correlation (Mahendran et al. 2024) and validated the suitability of PCA for assessing the overall quality of CTLs. The analysis retained the first four principal components (cumulative variance explained: 81.2%), selected based on the standard eigenvalue threshold > 1 (Table S5. This indicated that these components captured most of the metabolic variation in CTLs. Z-score standardization was used to the matrix of 13 characteristic variables. Principal component scores were then calculated using the component coefficient matrix and standardized data (Table S6, Fig. 7A), representing cigar tobacco characteristics (CTCs). Both EA and fermentation time had significant effects on CTLs. Specifically, scores of CTLs during day 10 and day 20 were higher than those at the unfermented stage (day 0). Except for the T1 group, which peaked at the end of fermentation (day 20), CTLs in the T0, T2 and T3 groups peaked at the mid-fermentation stage (day 10). CTLs fermented with EA showed significantly higher scores than T0. These results further confirmed the feasibility of EA in enhancing the industrial fermentation quality of CTLs. Further analysis revealed that microbial diversity, richness and dominant genera (top 10 in relative abundance) had both direct and indirect impacts on CTCs. These results were supported by acceptable model validation. Among these factors, bacterial influence was more pronounced than that of fungi (Fig. 7B and C). Bacterial diversity and richness had negative effects on CTCs (*p* < 0.05), whereas bacterial community composition showed a positive correlation (*p* < 0.05). A statistically significant positive correlation was observed between the relative abundance of dominant bacterial genera and overall bacterial diversity indices. (*p* < 0.05). In contrast, a significant inverse association was identified between CTC and fungal richness (*p* < 0.05). Bacterial diversity and fungal richness were found to be the main influencing factors when analyzing the overall effects of various indicators on CTCs (Fig. 7D and E). Notably, CTCs were consistently negatively impacted by microbial richness. Higher richness may lead to increased microbial competition for essential nutrients on the leaf surface, such as sugars and amino acids, which were crucial for cigar flavor, aroma and combustion properties.

## Conclusion

In conclusion, this study mainly compared and analyzed the changes of CTLs fermented with EA in terms of chemical composition, FAAs, VFCs and microbial communities. Compared to T0, the CTLs fermented with exhibited the higher reducing sugar content and lower levels of total sugar, starch, and protein. Meanwhile, EA facilitated nicotine degradation and promoted the accumulation of aroma compounds in CTLs, significantly enhancing the aroma profile of CTLs. Furthermore, EA altered the relative abundance of dominant microorganisms in CTLs. Co-occurrence network analysis revealed that EA particularly in T2 and T3 strengthened the cooperative interactions among the dominant microbial communities, thereby improving the stability of the microbial structure. According to the PLS-SEM model, EA might alter the microbial community assembly by reducing the total sugar content, thereby endowing CTLs fermented with EA with unique flavor characteristics. Furthermore, PCA verified the enhancing effects of EA on CTCs, and emphasized that this enhancing effects might be achieved by influencing bacterial alpha diversity and the formation of dominant communities. These research results provided a theoretical basis for the application of CHM in CTLs fermentation. Future studies will focus on investigating the effects of EA on CTLs from diverse geographical origins, optimizing critical fermentation parameters, and establishing a standardized technical framework. These efforts would provide a theoretical foundation for enhancing the industrial utilization of low-grade CTLs and developing distinctive fermentation techniques specific to CTLs.

## Supplementary Information

Additional file1 (XLSX 2306 KB)

## Data Availability

The datasets supporting the conclusions of this article are included within the article and its additional files.

## Abbreviations

CTLs: Cigar tobacco leaves
EA: Exogenous additives
CHM: Chinese herbal medicines
FAA: Free amino acids
VFCs: Volatile flavor compounds
VIP: Variable importance in the projection
OAV: Odor activity value
LEfSe: Linear discriminant analysis effect size
CTLs: Cigar tobacco characteristics
HS-SPME-GC-MS: Headspace Solid-Phase Microextraction coupled with Gas Chromatography-Mass Spectrometry
RI: Retention indices
OTUs: Operational Taxonomic Units
ANOVA: One-way analysis of variance
PCA: Principal component analysis
PLS-DA: Partial least-squares discriminant analysis
LDA: Linear discriminant analysis
TAV: Taste activity values
NMDS: Non-metric multidimensional scaling
PCoA: Principal coordinates analysis
PLS-SEM: Partial least squares structural equation modeling

## References

[CR1] Aboytes-Ojeda M, Castillo-Villar K, Yu T-h, Boyer C, English B, Larson J, Kline L, Labbé N (2016) A principal component analysis in switchgrass chemical composition. Energies. 10.3390/en9110913

[CR2] Bahk J, Marth EH (1983) Aflatoxin production is inhibited by selected herbal drugs. Mycopathologia 83:129–134. 10.1007/BF00437018

[CR3] Bian X, Miao W, Zhao M, Zhao Y, Xiao Y, Li N, Wu J-L (2022) Microbiota drive insoluble polysaccharides utilization via microbiome-metabolome interplay during Pu-erh tea fermentation. Food Chem 377:132007. 10.1016/j.foodchem.2021.132007 34999465 10.1016/j.foodchem.2021.132007

[CR4] Bolger AM, Lohse M, Usadel B (2014) Trimmomatic: a flexible trimmer for Illumina sequence data. Bioinformatics 30:2114–2120. 10.1093/bioinformatics/btu170 24695404 10.1093/bioinformatics/btu170 PMC4103590

[CR5] Cai B, Geng Z, Gao H, Lin B, Xing L, Hu X, Liu H (2019) Research progress of production technologies of cigar tobaccos in China. Acta Tab Sin 25:110–119. 10.16472/j.chinatobacco.2019.T00013

[CR6] Cai W, Feng T, Yao L, Sun M, Song S, Wang H, Yu C, Liu Q (2024) Characterisation of differential aroma markers in roasted coffee powder samples by GC×GC- TOF- MS and multivariate statistical analysis. Food Biosci 59:104207. 10.1016/j.fbio.2024.104207

[CR7] Chen Q, Li Z, Wang H, Huang YH, Cai L, Xie H, Zhou H (2019) Fungal composition and diversity of tobacco phyllosphere from cured tobacco leaves. Acta Microbiol Sin 59:2401–2409. 10.13343/j.cnki.wsxb.20190065

[CR8] Chen J, Han X, Wu Y, Liu L, Yu J, Li J, Zhang Y, Xu Y (2022) Comparative study on characteristics of mandarin peel extracts by biological processing. Arch Microbiol 204:512. 10.1007/s00203-022-03124-0 35864423 10.1007/s00203-022-03124-0

[CR9] Chen S, Zhu F, Zhang S, Wang S, Shen Y, Zhang M, Hu W, He Q, Qiu L, Hao Q, Li Z, Liu Z, Ding Y, Xu M, Kan H, Hu Y, Zhao X (2025) Integrated analysis of proteome and metabolome reveals the basis of amino acid metabolism in cigar artificial fermentation. Appl Biochem Biotechnol. 10.1007/s12010-025-05275-4 40434605 10.1007/s12010-025-05275-4

[CR10] Costa OYA, de Hollander M, Pijl A, Liu B, Kuramae EE (2020) Cultivation-independent and cultivation-dependent metagenomes reveal genetic and enzymatic potential of microbial community involved in the degradation of a complex microbial polymer. Microbiome 8:76. 10.1186/s40168-020-00836-7 32482164 10.1186/s40168-020-00836-7 PMC7265232

[CR11] Fu K, Song X, Cui Y, Zhou Q, Yin Y, Zhang J, Zhou H, Su Y (2024) Analyzing the quality differences between healthy and moldy cigar tobacco leaves during the air-curing process through fungal communities and physicochemical components. Front Microbiol 15:1399777. 10.3389/fmicb.2024.1399777 38887717 10.3389/fmicb.2024.1399777 PMC11180791

[CR12] Guo M, Wu F, Hao G, Qi Q, Li R, Li N, Wei L, Chai T (2017) *Bacillus subtilis* improves immunity and disease resistance in rabbits. Front Immunol 8:354. 10.3389/fimmu.2017.00354 28424690 10.3389/fimmu.2017.00354 PMC5372816

[CR13] Guo S, Li Y, Yang Z, Zhang Q, Li P, Jiang Z, Zhang J, Cao Y, Zhang Z, Li D (2024) Isolation and evaluation of Cyberlindnera fabianii strains to improve cigar tobacco leaves fermentation effect. Front Microbiol. 10.3389/fmicb.2024.1492042 39720475 10.3389/fmicb.2024.1492042 PMC11666510

[CR14] He Z, Can L, Wen Y, Sun M, Zhao H, Liu J (2025) Aroma characteristics and differential constituents analysis of cigar tobacco of different varieties and parts. J Agric Sci Technol. 10.1330/j.nykjdb.2024.0718

[CR15] Hu W, Cai W, Li D, Zheng Z, Liu Y, Luo C, Xue F (2023) Influence of fermentative medium on the chemical compositions and microbial communities of cigar tobacco leaves. J Light Ind 38:90–100 10.1038/s41598-022-23419-y PMC9649726 36357535

[CR16] Hu W, Cai W, Jia Y, Zhang Q, Zhang Z, Wang Y, Sun C, Li D (2024a) Fermentation of cigar tobacco leaves with citrus flavonoids: changes in chemical, microbiological, and sensory properties. Front Bioeng Biotechnol. 10.3389/fbioe.2024.1469532 39717530 10.3389/fbioe.2024.1469532 PMC11663678

[CR17] Hu Y, Zhao M, Qiu Y, Ye D, Liu Y, Zhang C, Wang H, Cheng J (2024b) Research progress on *dendrobii officinalis* caulis as medicinal and edible traditional Chinese medicine. J Nanjing Univ Chin Med 40:94–108. 10.14148/j.issn.1672-0482.2024.0094

[CR18] Hu W, Cai W, Liu J, Li M, Chen Q, Jia Y, Zhang Q, Li D (2025) Effects of active compounds extracted from natural spices on the quality of cigar tobacco leaves during fermentation. Chem Biol Technol Agric 12:69. 10.1186/s40538-025-00788-w

[CR19] Huang S, Liu D, Chen M, Xi G, Yang P, Jia C, Mao D (2022) Effects of *Bacillus subtilis* subsp. on the microbial community and aroma components of flue-cured tobacco leaves based on metagenome analysis. Arch Microbiol 204:726. 10.1007/s00203-022-03347-1 36427112 10.1007/s00203-022-03347-1

[CR20] Ji C, Li W, Yao L, He X, Luo D, Han T, He C, Li X (2025) Impact of various fertilization types and application rates on Salvia miltiorrhiza quality and the composition of its root microbial community. Ind Crops Prod. 10.1016/j.indcrop.2025.121071

[CR21] Jia Y, Zhou W, Yang Z, Zhou Q, Wang Y, Liu Y, Jia Y, Li D (2023) A critical assessment of the Candida strains isolated from cigar tobacco leaves. Front Bioeng Biotechnol 11:1201957. 10.3389/fbioe.2023.1201957 37691904 10.3389/fbioe.2023.1201957 PMC10485251

[CR22] Jiang TA (2019) Health benefits of culinary herbs and spices. J AOAC Int 102:395–411. 10.5740/jaoacint.18-0418 30651162 10.5740/jaoacint.18-0418

[CR23] Jiang C (2025) Differential analysis of of key aroma compounds in cigar filler tobacco leaves from different growing regions. Zhengzhou Tobacco Research Institute of CNTC, Retrieved from https://link.cnki.net/doi/10.27972/d.cnki.gzyzy.2025.000009

[CR24] Lei Z, Zehua L, Mingchuan Y, Shigui L, Yuhua X, Bin C, Haobao L, Dailong C, Jingang G, Bihua D (2021) Diversity of fermentation microbes and changes of hydrolytic enzyme activities of cigar leaf raw materials. J Agric Sci Technol 23:171–180. 10.13304/j.nykjdb.2020.0534

[CR25] Li N, Zhang C (2016) Effects of different materials on aroma components and sensory quality of cigar tobacco after fermentation. South China Agric 10:254–256. 10.19415/j.cnki.1673-890x.2016.03.152

[CR26] Li J, Wang J, Li J, Liu D, Li H, Gao W, Li J, Liu S (2016) *Aspergillus niger* enhance bioactive compounds biosynthesis as well as expression of functional genes in adventitious roots of *Glycyrrhiza uralensis* Fisch. Appl Biochem Biotechnol 178:576–593. 10.1007/s12010-015-1895-5 26490378 10.1007/s12010-015-1895-5

[CR27] Li J, Zhao Y, Qin Y, Shi H (2020) Influence of microbiota and metabolites on the quality of tobacco during fermentation. BMC Microbiol 20:356. 10.1186/s12866-020-02035-8 33213368 10.1186/s12866-020-02035-8 PMC7678276

[CR28] Li L, Mao Y, Yu J, Chen X, Yang C, Yao L (2023) Effects of bacillus megaterium on quality and bacterial community of cigar tobacco leaves. Human Agric Sci. 10.1649/j.cnki.hnnykx.2023.011.014

[CR29] Liu Z, Li X, Sun Z, Wang Z, Li G (2021) *Papiliotrema flavescens* colonized in biochars inhibits wheat crown rot and Fusarium head blight. Biochar 3:625–639. 10.1007/s42773-021-00121-2

[CR30] Luo H, Vong CT, Chen H, Gao Y, Lyu P, Qiu L, Zhao M, Liu Q, Cheng Z, Zou J, Yao P, Gao C, Wei J, Ung COL, Wang S, Zhong Z, Wang Y (2019) Naturally occurring anti-cancer compounds: shining from Chinese herbal medicine. Chin Med 14:48. 10.1186/s13020-019-0270-9 31719837 10.1186/s13020-019-0270-9 PMC6836491

[CR31] Ma L, Wang Y, Wang X, Lü X (2023) Solid-state fermentation improves tobacco leaves quality via the screened *bacillus subtilis* of simultaneously degrading starch and protein ability. Appl Biochem Biotechnol 196:506–521. 10.1007/s12010-023-04486-x 37148443 10.1007/s12010-023-04486-x

[CR32] Mahendran R, Abiharini S, Subbaraj A (2024) Unveiling the YouTube addiction: understanding the spectrum of digital dependency. J Fam Med Prim Care 13:5265–5269. 10.4103/jfmpc.jfmpc_1107_24 10.4103/jfmpc.jfmpc_1107_24 PMC11668467 39722913

[CR33] Ning Y, Zhang L, Mai J, Su J, Cai J, Chen Y, Jiang Y, Zhu M, Hu B (2023) Tobacco microbial screening and application in improving the quality of tobacco in different physical states. Bioresour Bioprocess 10:32. 10.1186/s40643-023-00651-6 38647749 10.1186/s40643-023-00651-6 PMC10992236

[CR34] Pei Q, Jiang X, Li Z, Xu H, Xie M, Xiong T, Liu Z (2025) Study on quality enhancement during cigar tobacco fermentation by *Staphylococcus nepalensis*: insights into microbial community, volatile substances and sensory evaluation. Front Microbiol 16:1526178. 10.3389/fmicb.2025.1526178 40008043 10.3389/fmicb.2025.1526178 PMC11850395

[CR35] Ren M, Qin Y, Zhao Y, Zhang B, Zhang R, Shi H (2024) Effects of microbes and metabolites on tobacco quality in"Humi"characteristic fermentation of cigar tobacco leaf. Process Biochem 143:186–197. 10.1016/j.procbio.2024.05.008

[CR36] Rönkkö M, Evermann J (2013) A critical examination of common beliefs about partial least squares path modeling. Organ Res Methods 16:425–448. 10.1177/1094428112474693

[CR37] Si H, Cui B, Liu F, Zhao M (2023) Microbial community and chemical composition of cigar tobacco (*Nicotiana tabacum* L.) leaves altered by tobacco wildfire disease. Plant Direct 7:e551. 10.1002/pld3.551 38099080 10.1002/pld3.551 PMC10719477

[CR38] Su Y, Cui Y, Fu K, Bu L, Sun Y, Zhou Q, Yin Y, Sun Y, Yang H, Wu L, Song X (2024) Contribution of pectin-degrading bacteria to the quality of cigar fermentation: an analysis based on microbial communities and physicochemical components. Front Microbiol 15:1481158. 10.3389/fmicb.2024.1481158 39611089 10.3389/fmicb.2024.1481158 PMC11604125

[CR39] Va VV, Xavier AS, David DCJB, Journal P (2019) In-vitro evaluation of antifungal and anticancer properties of *Tagetes erecta* petal extract. Biomed Pharmacol J 12:815–823. 10.13005/bpj/1705

[CR40] Wang L, Ning T, Chen X (2019) Postharvest storage quality of citrus fruit treated with a liquid ferment of Chinese herbs and probiotics. Sci Hortic 255:169–174. 10.1016/j.scienta.2019.03.030

[CR41] Wang Y, Luo X, Chu P, Shi H, Wang R, Li J, Zheng S (2023) Cultivation and application of nicotine-degrading bacteria and environmental functioning in tobacco planting soil. Bioresour Bioprocess 10:10. 10.1186/s40643-023-00630-x 38647817 10.1186/s40643-023-00630-x PMC10992035

[CR42] Wang H, Guo D, Ding N, Zhang M, Shi Y, Wu X, Mu D, Li X (2024a) Characterization of microbial communities, free amino acids and volatile flavor compounds in cigar tobacco leaves from Yunnan province. J Light Ind 39:97–108

[CR43] Wang H, Guo D, Zhang M, Wu G, Shi Y, Zhou J, Ding N, Chen X, Li X (2024b) Correlation study on microbial communities and volatile flavor compounds in cigar tobacco leaves of diverse origins. Appl Microbiol Biotechnol. 10.1007/s00253-024-13032-6 38407656 10.1007/s00253-024-13032-6 PMC10896874

[CR44] Wei R, Qiu F, Kong W, Wei J, Yang M, Luo Z, Qin J, Ma X (2013) Co-occurrence of aflatoxin B1, B2, G1, G2 and ochrotoxin A in *Glycyrrhiza uralensis* analyzed by HPLC-MS/MS. Food Control 32:216–221. 10.1016/j.foodcont.2012.11.028

[CR45] Wu T, Cheng D, He M, Pan S, Yao X, Xu X (2014) Antifungal action and inhibitory mechanism of polymethoxylated flavones from *Citrus reticulata* Blanco peel against *Aspergillus niger*. Food Control 35:354–359. 10.1016/j.foodcont.2013.07.027

[CR46] Wu Y, Zhao Z, Wu B, Zhang P, Liang S, Liu H (2019) Study on *dendrobium officinaleganoderma iucidum* wine with enhanced immunity. Food Res Dev 40:115–118

[CR47] Wu X, Hu Y, Wang Q, Liu J, Fang S, Huang D, Pang X, Cao J, Gao Y, Ning Y (2023) Study on the correlation between the dominant microflora and the main flavor substances in the fermentation process of cigar tobacco leaves. Front Microbiol. 10.3389/fmicb.2023.1267447 38075898 10.3389/fmicb.2023.1267447 PMC10699171

[CR48] Wu G, Zhang M, Han P, Guo D, Shi Y, Mu D, Li X, Wu X (2024a) Microbial community succession patterns and metabolite profiles in cigar tobacco during different mildew stages. Ind Crops Prod 222:120005. 10.1016/j.indcrop.2024.120005

[CR49] Wu G, Zhang M, Liu L, Wang H, Guo D, Shi Y, Mu D, Li X, Wu X (2024b) Mildew invasion: deciphering its influence on primary metabolites and microbial dynamics in fermented cigar tobacco ecosystems. Process Biochem 146:128–139. 10.1016/j.procbio.2024.07.004

[CR50] Xing Y, Chu F, Li J, Yao Y, Li Y, Yang Y, Bai W (2023) Study on bacteriostasis and feed mildew inhibition of 13 kinds of Chinese herbal medicine extracts. J Hainan Normal Univ (Nat Sci) 36:192–197. 10.12051/j.issn.1674-4942.2023.02.012

[CR51] Xu Y, Zhao J, Liu X, Zhang C, Zhao Z, Li X, Sun B (2022) Flavor mystery of Chinese traditional fermented baijiu: the great contribution of ester compounds. Food Chem 369:130920. 10.1016/j.foodchem.2021.130920 34461518 10.1016/j.foodchem.2021.130920

[CR52] Yan S, Ren T, Wan Mahari WA, Feng H, Xu C, Yun F, Waiho K, Wei Y, Lam SS, Liu G (2022) Soil carbon supplementation: improvement of root-surrounding soil bacterial communities, sugar and starch content in tobacco (*N. tabacum*). Sci Total Environ. 10.1016/j.scitotenv.2021.149835 34461468 10.1016/j.scitotenv.2021.149835

[CR53] Yang R, Yang J, Yu J, Wang S, Yang C, Xu F (2022) Effects of different nitrogen application rates on the quality and metabolomics of cigar tobacco. Agron J 114:1155–1167. 10.1002/agj2.20983

[CR54] Yun J, Qianying Z, Pinhe L, Wen C, Cheng L, Yuanfa L, Dongliang L (2023) Effects of constant and variable temperature fermentation on the quality of cigar tobacco. China Brew 42:212–217. 10.11882/j.issn.0254-5071.2023.06.034

[CR55] Zhang M, Shi H, Bi Y, Liu B, Zhou J, Duan W (2018) Changes of main ketone aroma components in burley, sun-cured and flue-cured tobaccos during 6 years in storage. Acta Tab Sin 24:23–24. 10.16472/j.chinatobacco.2018.122

[CR56] Zhang Q, Geng Z, Li D, Ding Z (2019) Characterization and discrimination of microbial community and co-occurrence patterns in fresh and strong flavor style flue-cured tobacco leaves. MicrobiologyOpen. 10.1002/mbo3.965 31808296 10.1002/mbo3.965 PMC7002102

[CR57] Zhang G, Li Z, Deng S, Li D, Zhang L, Cai B, Xiang X, Wang J, Wang F, Chen G, Zhang H, Liu H (2021) Characterization and succession analysis of bacterial community diversity in different fermentation cycles of Hainan H382 cigar leaf. Acta Tab Sin 26:117–126. 10.16472/j.chinatobacco.2020.170

[CR58] Zhang M, Liu Q, Pan Y, Zhang Y, Zhang X, Liang J, Wang H, Zhang X, Wu Y, Fu B, Zou L (2023a) Screening and identification of tannin-degrading bacteria to optimize fermentation of cigar tobacco. Tobacco Sci Technol 56:32–40. 10.16135/j.issn1002-0861.2023.0091

[CR59] Zhang W, Yang X, Zhang Q, Zhu W, Lu Y, Shang X (2023b) Effect of exogenous neutral protease fermentation on cigar leaf quality. BIO Web of Conferences 60:01020. 10.1051/bioconf/20236001020

[CR60] Zhang X, Miao Q, Pan C, Yin J, Wang L, Qu L, Yin Y, Wei Y (2023c) Research advances in probiotic fermentation of Chinese herbal medicines. iMeta. 10.1002/imt2.93 38868438 10.1002/imt2.93 PMC10989925

[CR61] Zhang L, Mai J, Shi J, Ai K, He L, Zhu M, Hu B (2024a) Study on tobacco quality improvement and bacterial community succession during microbial co-fermentation. Ind Crops Prod. 10.1016/j.indcrop.2023.117889

[CR62] Zhang M, Guo D, Wang H, Wu G, Ding N, Shi Y, Zhou J, Zhao E, Li X (2024b) Integrated characterization of filler tobacco leaves: HS–SPME–GC–MS, E-nose, and microbiome analysis across different origins. Bioresour Bioprocess 11:11. 10.1186/s40643-024-00728-w 38647645 10.1186/s40643-024-00728-w PMC10992047

[CR63] Zhang M, Guo D, Wang H, Wu G, Shi Y, Zhou J, Zheng T, Zhao E, Wu X, Li X (2024c) Comparative profiling of microbial communities and volatile organic compounds in fermented wrapper, binder, and filler cigar tobaccos. Chem Biol Technol Agric 11:68. 10.1186/s40538-024-00582-0

[CR64] Zhang Q, Huang Y, An H, Yang S, Lei J, Wang Y, Li P, Zhang H, Cai W, Jia Y, Pang Y, Li D (2024d) The impact of gradient variable temperature fermentation on the quality of cigar tobacco leaves. Front Microbiol 15:1433656. 10.3389/fmicb.2024.1433656 39735193 10.3389/fmicb.2024.1433656 PMC11672604

[CR65] Zhang Y, Waghmare PR, Zhang Z, Gao L (2024e) Co-production of sugars and aroma compounds from tobacco waste using biomass-degrading enzymes produced by *Aspergillus brunneoviolaceus* Ab-10. Arch Microbiol 206:291. 10.1007/s00203-024-03972-y 38849576 10.1007/s00203-024-03972-y

[CR66] Zhang L, Li W, Peng Z, Zhang J (2025) Effect of microbial community on the formation of flavor components in cigar tobacco leaves during air-curing. BMC Microbiol 25:56. 10.1186/s12866-025-03774-2 39891085 10.1186/s12866-025-03774-2 PMC11783773

[CR67] Zhao X, Yun J-m, Ai D-y, Zhang W-w, Zhao F-q, Li H-z, Jia Y-l (2016) Effects of four kinds of Chinese herbs extracts on ganoderma triterpenoids production of *Ganoderma japonicum* in submerged fermentation. Food Ferment Ind 42:97–103. 10.13995/j.cnki.11-1802/ts.201603017

[CR68] Zhou H, Xu S, Xu B, Jiang C, Zhao E, Xu Q, Hong J, Li X (2024) Effect of *Caproicibacterium lactatifermentans* inoculation on the microbial succession and flavor formation of pit mud used in Chinese Baijiu fermentation. Food Res Int 175:113730. 10.1016/j.foodres.2023.113730 38129040 10.1016/j.foodres.2023.113730

[CR69] Zhou H, Yang Y, Jia T, Yu Y, Chen S, Qiu Y, Zhang R, Chen H (2025a) Controlling mildew of tobacco leaf by *Bacillus amyloliquefaciens* ZH-2 and its effect on storage quality of tobacco leaf. Sci Rep 15:5304. 10.1038/s41598-025-90058-4 39939685 10.1038/s41598-025-90058-4 PMC11821844

[CR70] Zhou M, Zhang Y, Song Z, Tang S, Liu Z, Pang M, Zhang D, Wu X, Yu X, Wang P, Wei Y (2025b) Enhanced bioactivity of honeysuckle-Cassia seeds extracts through Lactobacillus acidophilus and Bacillus subtilis co-fermentation: Impact on alcoholic liver disease and gut microbiota. Food Chem. 10.1016/j.foodchem.2025.144463 40339419 10.1016/j.foodchem.2025.144463

[CR71] Zhuang J, Xiao Q, Feng T, Huang Q, Ho C-T, Song S (2020) Comparative flavor profile analysis of four different varieties of *Boletus* mushrooms by instrumental and sensory techniques. Food Res Int 136:109485. 10.1016/j.foodres.2020.109485 32846567 10.1016/j.foodres.2020.109485

